# Mechanism-Based Pharmacokinetic/Pharmacodynamic Model of Voriconazole for Predicting the Clinical Outcomes of Adult Patients With Invasive Aspergillosis

**DOI:** 10.1097/FTD.0000000000001268

**Published:** 2024-10-22

**Authors:** Monchai Duangpraphat, Richard C. Wilson, Timothy M. Rawson, Wichai Santimaleeworagun, Worapong Nasomsong, Alison H. Holmes, Vasin Vasikasin

**Affiliations:** * Department of Internal Medicine, Phramongkutklao Hospital and College of Medicine, Ratchadhevi, Bangkok, Thailand;; † Centre for Antimicrobial Optimisation, Imperial College London, United Kingdom;; ‡ David Price Evans Global Health and Infectious Diseases Research Group, University of Liverpool, United Kingdom;; § NIHR Health Protection Research Unit in Healthcare Associated Infections and Antimicrobial Resistance, Imperial College London, Hammersmith Hospital, London, United Kingdom; and; ¶ Department of Pharmaceutical Care, Faculty of Pharmacy, Silpakorn University, Nakorn Pathom, Thailand.

**Keywords:** voriconazole, pharmacokinetic–pharmacodynamic model, galactomannan, invasive aspergillosis

## Abstract

**Background:**

Voriconazole is the first-line therapy for invasive aspergillosis (IA). To determine the minimum inhibitory concentration of Aspergillus, a voriconazole pharmacokinetic–pharmacodynamic (PK–PD) model linked to galactomannan response was developed and evaluated, and its clinical correlation for IA treatment was elucidated.

**Methods:**

Adult patients with probable or definite IA and at least one serum voriconazole measurement were included. A two-compartment voriconazole PK model was linked to a previously described PD model of galactomannan response. PK and PD parameters were estimated using a nonparametric adaptive grid technique. The relationship between the ratio of voriconazole exposure that induced half-maximum galactomannan response (EC50) and the observed terminal galactomannan concentration was evaluated. The factors associated with the PK–PD parameters and mortality were also determined.

**Results:**

Between January 2013 and December 2022, 41 patients were prescribed voriconazole for IA. The 30-day mortality rate was 17%. A high correlation was found for the observed–predicted Bayesian posterior estimates of voriconazole and galactomannan levels. Moreover, a nonlinear relationship was identified between AUC:EC50 and terminal galactomannan. The factors associated with higher AUC:EC50 were intravenous administration and intubation. In the survival analysis, higher EC50 tended to be associated with mortality, higher AUC was significantly associated with increased mortality, and higher AUC:EC50 tended to be associated with higher mortality. After adjusting for the intravenous route, higher AUC and AUC:EC50 were not associated with mortality.

**Conclusions:**

Individual EC50 estimation can provide insights into in vivo host and organism responses. Elevated EC50 showed comparable and unfavorable trends to higher minimum inhibitory concentration. Thus, determining EC50 might help guide individualized target serum voriconazole levels.

## Abstract 2

Supplemental Digital Content is Available in the Text.

## INTRODUCTION

Voriconazole is the first-line antifungal for the management of invasive aspergillosis (IA) across all age groups.^1–3^ However, considerable interindividual variance in the voriconazole pharmacokinetics (PK) is observed, thereby warranting the application of therapeutic drug monitoring (TDM) to optimize dosing strategies.^1–3^

Traditional approaches for estimating voriconazole pharmacodynamics (PD) use the minimum inhibitory concentration (MIC) assessment. For voriconazole, the ratio of the area under the unbound drug concentration–time curve to the MIC (AUC:MIC) has been linked to clinical response.^4^ However, AUC:MIC is associated with notable drawbacks, including challenges isolating *Aspergillus* spp. from clinical specimens.^5^ Even when feasible, antifungal susceptibility testing is not universally available, has long turnaround times, and lacks interpretive criteria for certain species.^6^ Consequently, the diagnosis of IA predominantly relies on the serum antigen, galactomannan.^1,3,5^

Galactomannan, a large molecule present on the fungal cell wall, is primarily detected in the bloodstream during fungal proliferation and dissemination. Monitoring serum galactomannan levels in high-risk individuals facilitates the diagnosis of IA and may aid in assessing response to antifungal therapy.^1,3,7^ Notably, persistently elevated antigen levels have been associated with unfavorable treatment outcomes.^8,9^

To overcome the challenges associated with MIC determination, a novel approach known as mechanism-based modeling has been proposed. This method leverages changes in clinical response indicators within the body to construct models, substituting MICs with a concentration set at the half maximal effective concentration (EC50) on a model fitted to the time–kill curve data.^10^ Mechanism-based PK–PD modeling, derived in vitro, holds promise as an effective tool for characterizing the efficacy of antimicrobial agents and is recommended for experimental and clinical study designs.^11^

An observational study with the pediatric population resulted in the development of a mechanism-based PK–PD model of voriconazole based on changes in serum galactomannan levels.^12^ Although the study suggested potential clinical correlations based on better PK–PD profiles in surviving patients, the limited sample size precluded the observation of significant improvements in clinical outcomes. Furthermore, investigations revealing the model efficacy in adult patients are lacking.

If the model could help predict clinical outcomes, voriconazole dosing may be optimized to enhance the treatment of IA. Therefore, we aimed to develop a PK–PD model using serum galactomannan levels in adults and determine the correlation between model outcomes and clinical responses.

## MATERIALS AND METHODS

### Study Design and Participants

We conducted a retrospective cohort study at Phramongkutklao Hospital, a 1200-bed tertiary care hospital in Bangkok, Thailand, from January 2013 to December 2022. Eligible participants were adults older than 18 years with voriconazole blood levels measured at least once, serum galactomannan measured at least once, and diagnosed with probable or proven IA according to the EORTC/MSG criteria.^7^ Ethical approval was obtained from the Institutional Review Board of the Royal Thai Army Medical Department [R072h/65]. Informed consent was waived due to the observational nature of the study.

### Specimen Collection

In routine clinical practice at the study site, the dosing regimen includes the administration of 6 mg/kg voriconazole every 12 hours during the first 24 hours followed by 4 mg/kg every 12 hours thereafter. The route of administration is determined by the physician. When orally administered, voriconazole is taken on an empty stomach.

Patients undergoing voriconazole treatment were advised to undergo TDM, with blood samples typically collected 3 days postadministration. The target trough concentration of voriconazole ranged from 1 mg/L to 6 mg/L. Voriconazole levels were monitored over the next few days, with adjustments made to medication dosages if concentrations deviate from the target range. Owing to the long turnaround time for obtaining the serum voriconazole results, adjustments were made 7 days postadministration.

Serum galactomannan levels were not used for medication adjustments. Voriconazole and galactomannan assays were conducted via liquid chromatography-tandem mass spectrometry (Acquity UPLC H-Class PLUS System/Xevo TQ-S micro IVD System, Waters Corp, Milford, MA) and enzyme immunoassay (Euroimmun Medizinische Labordiagnostika, Lübeck, Germany), respectively. The voriconazole assay had a limit of quantification of 0.05 mcg/mL, with accuracy and precision (%CV) of 87.8% and 7.9%, respectively.^13^

### PK–PD Modeling

PK–PD modeling integrated PK (voriconazole) and PD (galactomannan concentration) data. The Pmetrics population program facilitated all modeling processes using a nonparametric adaptive grid technique.^14^ Mathematical equations were employed to describe the rate of change of voriconazole and galactomannan levels in various compartments within the body, aligning with previous methodologies by Huurneman et al.^12^ In brief, the model consisted of 3 differential equations for the rate of change of voriconazole within each compartment, and another equation for the rate of change of serum galactomannan.

First, PK parameters were estimated using a previously established population PK model in the adult population.^15^ The structural equations were as follows:

(1) $$\frac{dX_{1}}{dt}=-K_{a}∙X_{1}$$

(2) $$\frac{dX_{2}}{dt}=K_{a}∙X_{1}+\text{RateIV}-\frac{V_{\max}}{K_{m}∙V+X_{2}}∙X_{2}-K_{cp}∙X_{2}+K_{pc}∙X_{3}$$

(3) $$\frac{dX_{3}}{dt}=K_{cp}∙X_{2}-K_{pc}∙X_{3}$$

where X(1) represents the amount of voriconazole (mg) in the gut compartment in patients administered an oral bolus dose, and Ka is the first-order rate constant of drug absorption.

X(2) and X(3) represent the amount of voriconazole (mg) in the central (c) and peripheral (p) compartments. RateIV is the rate of infusion of voriconazole into the central compartment (mg/h). Vmax is the maximum rate of enzyme activity in the metabolism of voriconazole (mg/h). V is the volume of the central compartment. Km is the concentration of voriconazole in the central compartment where voriconazole clearance is half maximal. The 2 compartments are connected by the first-order rate constants, Kcp and Kpc (h-1).

Bayesian posterior estimates for the PK parameters of each patient were fixed, and the PD parameters were estimated by fitting the PD component of the model to individual galactomannan data. The PD model was as follows:

(4a) $$\frac{dX_{4}}{dt}={KGM}_{prod}∙\left[1-\left(\frac{x_{4}}{{POP}_{\max}}\right)\right]∙\left(1-\frac{\frac{X_{2}^{H}}{V}}{{EC}_{50}^{H}+\frac{X_{2}^{H}}{V}}\right)∙X_{4}$$

(4b) $$-{KGM}_{e\;\lim}∙X_{4\;}$$

KGMprod is the maximum rate of galactomannan production. POPmax is the maximum value of galactomannan. KGMelim is the rate of maximal galactomannan elimination, H is the slope function for galactomannan elimination, and EC50 is the concentration of voriconazole (mg/L) that produces the half maximal effect on galactomannan reduction.

Coefficients of determination (R^2^) from a linear regression of the observed–predicted Bayesian posterior estimates were used to assess the fit of the model.

Exposure–response relationships were evaluated using the AUC of voriconazole to EC50. To minimize selection bias caused by the inclusion of only patients who survived long enough to have their doses adjusted based on the TDM results, only the AUC of the first 168 hours (7 days) was used. The AUC was averaged daily over this initial treatment period.

All-cause mortality within 30 days of voriconazole initiation was the primary outcome measure. Secondary outcomes included the length of hospital stay and duration of ventilator use.

### Statistical Analysis

The sample size calculation was based on a prior study by Huurneman et al.^12^ Among the 12 participants, 4 fatalities were recorded in the group with AUC:EC50 above the median, representing 66.6%, and 6 fatalities were recorded in the group with model values below the median, representing 100%. With a power of 0.8 and type one error of 0.025 for determining the superiority of patients with AUC:EC50 value above the median, a sample size of at least 32 patients was needed.

Statistical analysis was performed using R, with descriptive statistics used to summarize the data characteristics. Statistical inference analysis determined significance at a 2-sided *P*-value <0.05. Group comparisons were conducted using unpaired *t* tests or Mann–Whitney *U* tests for continuous variables and χ^2^ tests or Fisher exact tests for proportions, as appropriate.

The relationship between AUC:EC50 and terminal galactomannan was assessed using exponential correlation. The test for correlation was conducted using Pearson correlation coefficient.

Univariate and multivariate Cox regression models were used to assess the relationship between PK–PD modeling parameters and mortality. The models included numeric values for EC50, AUC, and AUC:EC50. To compare patients with high and low AUC:EC50 and EC50, Kaplan–Meier survival curves were generated. The parameters were categorized into 2 groups based on whether the values were below or above the median levels.

## RESULTS

Between January 2013 and December 2022, 125 patients were prescribed voriconazole. A total of 84 cases were excluded, including 35 pediatric cases, 28 cases without voriconazole levels, 12 cases without IA diagnosis, and 9 cases of possible IA. Thus, a total of 41 patients with probable (35/41, 85.4%) or proven (6/41, 14.6%) IA were included. The demographic characteristics are summarized in Table 1. Most patients were males (25/41, 61.0%), and hematological malignancy (29/41, 70.7%) was the most common indication. A total of 15 patients had neutropenia, defined as absolute neutrophil count <0.5 × 10^9^/L, at the day of diagnosis (36.6%). Amphotericin B was prescribed to 31 patients before voriconazole (75.6%). Intravenous voriconazole was prescribed to 12 patients (29.3%). The 30-day mortality rate was 17.1% (7/41). A combination of antifungals was not prescribed.

**TABLE 1.** Patients Characteristics and Outcomes Divided Into 2 Groups: Higher AUC:EC50 and Lower AUC:EC50 Groups

Characteristic	Total, n = 41	Higher AUC:EC50, n = 19	Lower AUC:EC50, n = 22	*P*
Male	25 (61.0)	13 (68.4)	12 (54.5)	0.3885
Age (yr)	53 (37–62)	52 (38–65)	55 (37.25–61.5)	0.8322
Weight (kg)	63 (54–68)	61 (52–69)	63 (59.25–67.75)	0.7777
Height (cm)	165 (160–173)	168 (161–172.5)	163 (158–173.8)	0.8931
Underlying diseases				
Malignancy	30 (73.2)	13 (68.4)	17 (77.3)	0.5480
ALL	4 (9.8)	2 (10.5)	2 (9.1)	
AML	18 (43.9)	7 (36.8)	11 (50.0)	
Lymphoma	6 (14.6)	4 (21.1)	2 (9.1)	
MM	1 (2.4)	0 (0.0)	1 (4.5)	
NSCLC stage 4	1 (2.4)	0 (0.0)	1 (4.5)	
Post kidney transplant	2 (4.9)	0 (0.0)	2 (9.1)	—
Post-COVID-19	2 (4.9)	0 (0.0)	2 (9.1)	—
Others*	8 (19.5)	7 (36.8)	1 (4.5)	—
Status on the first day of voriconazole				
Creatinine clearance (mL/min)	62.2 (40.9–81.7)	58.3 (38.5–72.3)	73.6 (46.1–100.6)	0.7476
Neutropenia†	15 (36.6)	9 (47.4)	6 (27.3)	0.2054
Shock	3 (7.3)	1 (5.3)	2 (9.1)	1.0000
Intubation	8 (19.5)	7 (36.8)	1 (4.5)	0.0157
Amphotericin B pretreatment	31 (75.6)	15 (78.9)	16 (72.7)	0.6683
Intervention				
Intravenous administration	12 (29.3)	9 (47.4)	3 (13.6)	0.0367
Amphotericin duration (d)	6 (0–16)	5 (0–15.5)	7 (0–16.25)	0.9842
Outcome				
30-day mortality	7 (17.1)	5 (26.3)	2 (9.1)	0.2190
Length of hospital stay (d)	47 (38–58)	50 (37–56.5)	46.5 (40.25–61.77)	0.5975
Ventilator day	12 (8.5–17.5)	10 (5–16.5)	15 (12.75–18.25)	0.8229

Higher AUC:EC50 was based on a value above the median of 216.

Data indicate n(%) or median(IQR).

* Others = hypersensitivity pneumonitis, pemphigus vulgaris, primary myelofibrosis, rheumatoid arthritis, lupus nephritis, and type 2 diabetes in the high AUC:EC50 group and psoriasis in the low AUC:EC50 group.

† Absolute neutrophil count <0.5 × 10^9^/L.

AML, acute myeloid leukemia; ALL, acute lymphoblastic leukemia; MM, multiple myeloma; NSCLC, nonsmall cell lung cancer.

**FIGURE 1.**
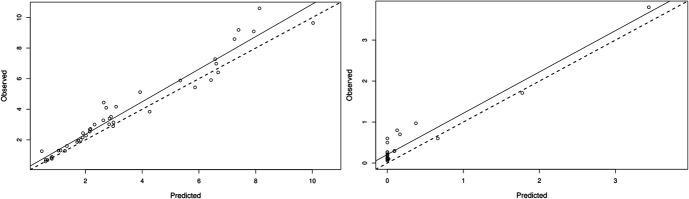
Linear regression results for the observed–predicted Bayesian posterior estimates of voriconazole (left) and galactomannan levels (right).

The linear regression results for observed–predicted Bayesian posterior estimates for the individual PK and PD of voriconazole and galactomannan levels are shown in Figure 1. A high correlation was found between the observed and predicted Bayesian posterior estimates of voriconazole and galactomannan concentrations (R^2^ = 0.95 and R^2^ = 0.93, respectively). Individual observed and predicted concentration–time profiles are presented in **Supplemental Digital Content 1** (see Figure S1, http://links.lww.com/TDM/A801).

**FIGURE 2.**
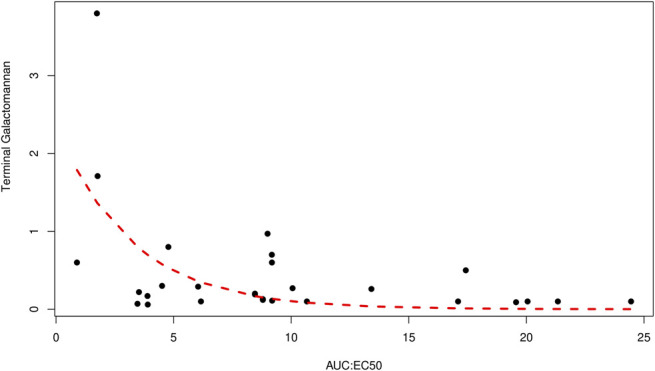
Correlation between AUC:EC50 and terminal galactomannan levels.

A nonlinear relationship was identified between AUC:EC50 and the terminal galactomannan (*P* < 0.001), as shown in Figure 2. Factors significantly associated with higher AUC:EC50, determined based on a value above the median of 216, were intravenous voriconazole administration (RR: 2.18, 95% CI: 1.20–3.96, *P* = 0.037) and intubation (RR: 2.41, 95% CI: 1.43–4.05, *P* = 0.016), as shown in Table 1. Mortality, length of hospital stay, and duration of ventilator use were not associated with the AUC:EC50 values.

**FIGURE 3.**
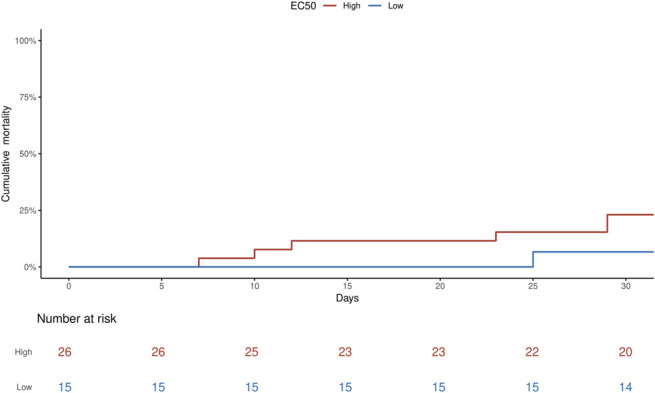
Cumulative mortality over a 30-day period among patients with high EC50 compared with those with low EC50.

In the survival analysis, higher EC50 tended to be associated with higher mortality (HR: 1.59, 95% CI: 0.50–5.02, *P* = 0.430). Figure 3 describes the comparison between probability of survival in patients with high EC50 (>0.48 mg/L) and low EC50 (mortality 23.1% vs. 6.7%, HR: 3.8, 95% CI: 0.46–32, *P* = 0.216). Higher AUC was significantly associated with increased mortality (HR: 1.31, 95% CI: 1.01–1.70, *P* = 0.038) and higher AUC:EC50 tended to be associated with higher mortality (HR: 1.11, 95% CI: 0.98–1.24, *P* = 0.089).

Other factors significantly associated with increased mortality included intravenous voriconazole administration, as shown in Table 2. After adjustment for intravenous administration, higher AUC and AUC:EC50 were not associated with mortality (HR: 1.20, 95% CI: 0.89–1.63, *P* = 0.239 and HR: 1.06, 95% CI: 0.92–1.21, *P* = 0.427, respectively).

**TABLE 2.** Factors Associated With 30-Day Mortality

Factor	HR (95% CI)	*P*	Adjusted HR (95% CI)	*P*
Male	0.78 (0.17–3.47)	0.741		
Age	0.99 (0.95–1.03)	0.572		
Weight	0.99 (0.93–1.06)	0.811		
Height	0.97 (0.89–1.06)	0.561		
Creatinine	0.45 (0.12–1.71)	0.243		
Malignancy	—	0.999		
Neutropenia	2.33 (0.52–10.4)	0.269		
Shock	2.80 (0.34–23.31)	0.341		
Intravenous administration	7.21 (1.4–37.21)	0.0184		
Intubation	1.97 (0.38–10.17)	0.418		
Amphotericin B pretreatment	2.02 (0.24–16.79)	0.515		
EC50	1.59 (0.5–5.02)	0.430	1.74 (0.53–5.64)	0.359
AUC	1.31 (1.01–1.7)	0.038	1.20 (0.89–1.63)	0.239
AUC:EC50	1.11 (0.98–1.24)	0.089	1.06 (0.92–1.21)	0.427

## DISCUSSION

In this study, we successfully developed a mechanism-based PK–PD model that had a strong correlation between individual predicted and observed levels. Notably, a nonlinear relationship was observed between the AUC:EC50 and terminal galactomannan levels. Intravenous administration and intubation were identified as factors associated with higher AUC:EC50.

The observed mortality rate of 17.1% was markedly lower than the 83.3% reported in a previous study.^12^ This difference may be attributed to differences in patient demographics and inclusion criteria; the present study exclusively comprised adults with proven or probable IA. A multicenter study reported that mortality rates are lower among the adult population than the pediatric population.^16^ In pediatric groups, the diagnosis of an invasive mold infection is challenging and is mainly determined by the extent of invasive disease and the severity of immunosuppression. The PK of voriconazole is highly variable in the pediatric population as the interindividual variability of voriconazole PK is larger in this cohort.^17^ Finally, our study included only patients with proven or probable IA, whereas the previous study included one-third of patients with possible IA.^12^ The inclusion of patients with possible IA in the previous study might have introduced confounding variables, such as concurrent infections, which could have contributed to elevated mortality rates.

Treatment conditions also differed between the 2 studies, with most patients in the prior study receiving amphotericin B before voriconazole administration, in adherence with Thai national guidelines that list voriconazole as a medication specifically for patients with proven or probable IA.

Less than 30% of patients received intravenous voriconazole, indicating that most patients could take oral medication and were not critically ill. Intravenous voriconazole administration was associated with higher AUC:EC50, which might be due to higher drug exposure induced by weight-based drug doses than that induced by fixed-doses established for oral administration. The study also identified a link between intubation and higher AUC:EC50 values. Thus, patients who were intubated had more severe illnesses, thereby warranting higher dosages or intravenous administration, as determined by the attending physician.

Our mechanism-based PK–PD model revealed a nonlinear relationship between AUC:EC50 and the terminal galactomannan, aligning with the results of previous studies.^9,12^ This finding helps highlight the true relationship between AUC:EC50 and galactomannan.

Higher AUC and AUC:EC50 ratios were significantly associated with increased mortality, whereas higher EC50 values tended to be associated with increased mortality. This association was not found after adjusting for other factors associated with mortality. Although the high estimate for EC50 may be due to various factors directly impacting mortality, such as a high fungal burden or antifungal resistance, AUC can be affected by factors, such as physicians preference in prescribing intravenous antifungal for critically ill patients. Therefore, higher AUC and AUC:EC50 were not associated with improved mortality in this study. These findings underscore the importance of various aspects in the treatment of IA in immunocompromised patients, suggesting that factors beyond PK–PD considerations may influence outcomes.

The smaller-than-expected dataset of serum voriconazole and galactomannan levels served as the main limitations of the study. This limitation highlights the need for future studies to gather more comprehensive datasets to further refine the PK–PD model.

In conclusion, individual EC50 estimation offers valuable insights into in vivo host and organism responses, complementing traditional MIC-based approaches. Although elevated EC50 values tended to be similar to higher MIC values, AUC:MIC ratios were not directly associated with mortality. Nonetheless, determining EC50 values may aid in tailoring individualized target serum voriconazole levels for optimal therapeutic outcomes.
